# Urethral pressure variation: a neglected contributing factor in patients with overactive bladder syndrome?

**DOI:** 10.1590/S1677-5538.IBJU.2016.0308

**Published:** 2017

**Authors:** Ruth Kirschner-Hermanns, Ralf Anding, Nariman Gadzhiev, Ing Goping, Adele Campbell, Nadine Huppertz

**Affiliations:** 1 Departament of Neuro-urology, University Hospital Friederich Wilhelms, University Bonn, Germany;

**Keywords:** Urinary Bladder, Overactive, Urodynamics, Urethra

## Abstract

**Objective:**

To study urethral pressure variations during the whole filling phase among different groups of patients.

**Material and Methods:**

We investigated 79 consecutive patients from January 2011 to June 2012. All patients were recruited within our routine practice in our continence clinic and were evaluated with urodynamic exam according to the standards of the International Continence Society (ICS) with an additional continuous measurement of the urethral pressure profile (cUPP) that was done in a supine position. Patients with genital prolapse >grade I, as well as patients with impaired cognitive function or neurogenic disorders were excluded. Bacteriuria at the time of investigation was excluded by urine analysis. Urethral pressure changes higher than 15cmH_2_O were considered as ‘urethral instability’.

**Results:**

From 79 investigated patients, 29 were clinically diagnosed with OAB syndrome, 19 with stress urinary incontinence (SUI) and 31 with mixed (OAB and SUI) incontinence. The prevalence of ‘urethral instability’ as defined in this study was 54.4% (43/79). The mean Δp in patients with OAB (36.5cmH2O) was significantly higher (p<0.05) than in groups with pure stress (14.9cmH2O) and mixed urinary incontinence (19.3cmH2O).

**Conclusions:**

Etiology of ‘urethral instability’ is unknown, but high prevalence among patients with overactive bladder syndrome, especially concomitant with detrusor activity can raise a fair question and direct further diagnostic as well as treatment efforts.

## INTRODUCTION

Overactive bladder (OAB) syndrome has a great impact on health-related quality of life with a high prevalence of up to 13%, in the female population over 18 years (1). According to the International Urogynecological Association (IUGA) and the International Continence Society (ICS), the term OAB describes the combination of symptoms consisting of urgency, with or without urge urinary incontinence, urinary frequency and nocturia, if there is no proven infection or other obvious pathological condition (2). But at the same time term OAB denotes a syndrome whose etiology is unknown, and it is believed that detrusor overactivity (DO) is a major factor and is currently the only accepted underlying pathophysiology of OAB (3). The fact that during classic urethral pressure profile measurement catheter withdrawal along the urethra gives only a little information concerning external urethral sphincter behavior during the whole filling phase, which is according to “guarding reflex” theory by Park et al. can have a bladder-modulating role (4), as well as low efficacy of the existing drugs (anti-muscarinic, beta3-mimetics) targeting at the detrusor wall (5) – all that encouraged us to look closer if there is any consistent pattern of external sphincter functioning in different group of patients. In this regard, we investigated urethral sphincter pressure variations and in OAB, SUI and MUI patient’s continuously measuring urethral pressure profile during the whole filling phase.

## MATERIALS AND METHODS

### Study Design

Ethical approval (EK 085/11-Universityclinic Aachen) and patient consent was obtained and studies were done in accordance to the declaration of Helsinki. The study evaluated 79 consecutive female patients with lower urinary tract symptoms (LUTS), including signs of overactive bladder syndrome (OAB), stress (SUI) and mixed urinary incontinence (MUI). Patients were referred to the Continence clinic of the University Hospital (RWTH) Aachen, an interdisciplinary unit for incontinence diagnostics. Period of enrollment was from January 2012 through June 2012.

Patients with genital prolapse >grade I, as well as patients with impaired cognitive function or neurogenic disorders were excluded. Bacteriuria at the time of investigation was excluded by urine analysis. All patients underwent pressure/flow studies followed by a conventional urethral pressure profile measurement with a triple lumen 9Fr catheter and a continuous urethral pressure profile (cUPP) registration for 60s during a second complete filling phase with the catheter positioned at the site of the maximum urethral closure pressure. The bladder was filled with medium filling speed of 15 to 30mL/min. The cUPP was done in a supine position to reduce movement artifacts and the patient was asked to lie relaxed without movement if possible. Throughout the investigation, pelvic floor electromyography (EMG) was registered by surface electrodes.

### Evaluation of cUPP

The difference between the highest and lowest urethral pressure during cUPP was calculated. A urethral pressure drop with urgency to void before micturition was neglected, because focus of analysis was on the cUPP during filling phase. Urethral pressure variations exceeding 15cmH_2_O were defined as ‘urethral instability’ (6-12). Urethral pressure variation (cmH_2_O), maximum urethral closing pressure (cmH_2_O), minimum urethral closing pressure (cmH_2_0) and functional urethral length were determined for each patient group (OAB, MUI, SUI).

### Area Under the Curve (AUC)

The 60s of raw data was obtained through the ASCII-exportation of Laborie UDS data into a standard spreadsheet program and included time and corresponding urethral pressure with a sample rate of 10Hz. Area under the curve (AUC) was calculated with the trapezoidal rule and corresponding minimum pressure values. Data were pooled for every patient group (OAB, MUI, SUI).

### Fast Fourier Transform (FFT)

Raw data were used for the application of Fast Fourier Transform (FFT) and graphic functions. Basic control data (eg: known sinusoidal data) was also used to validate the FFT process to ensure that the frequency domain transformation was correct. Applying a Fast Fourier Transform (FFT) converted the temporal information of P_ura_ into the frequency domain and thereby allowed the reviewer to visualize, from the spectral density perspective, the frequency content of the signal under review. While no specific frequency component was expected to be identified during a ‘urethral instability’, we were looking for large clusters of frequency content to be uniquely differentiated from normal P_ura_ measurements. The FFT samples were calculated and plotted for a visual review.

### Statistics

Statistical analysis was performed using NCSS (NCSS, LLC release 2007, Utah, USA) and GraphPad Prism Ver. 5 (GraphPad Software Inc., La Jolla, CA, USA). Data were analyzed using the Shapiro-Wilk normality test and non-parametric Mann-Whitney u-test. Relationship between ‘urethral instability’ and functional urethral length was quantified by Spearman-rank test and Pearson r-squared correlation. A p-value of <0.05 was considered statistically significant. All statistical tests reported in this study were two tailed.

## RESULTS

Of the 79 female patients, 29 presented with OAB syndrome, 19 with SUI and 31 with mixed (OAB and SUI) symptoms. Patient’s characteristics are shown in Table-1.

**Table 1:** Main patient’s characteristics.

Diagnosis	n	Age (years) (mean±SD)	Urethral pressure variation (cm H_2_0)	Maximum urethral closing pressure (cm H_2_0)	Minimum urethral closing pressure (cm H_2_0)	Functional urethral length (mm)	Post Menop. %
OAB	29	60.2±13.6	36.5±28.3	202.1	1.0	38.0±7.9	79.3
Stress incontinence	19	62.8±12.1	14.9±6.6	114.5	3.0	33.9±8.7	84.2
Mixed incontinence	31	58.5±14.4	19.3±10.47	89.0	3.8	32.1±6.6	77.4

**Figure 1:**
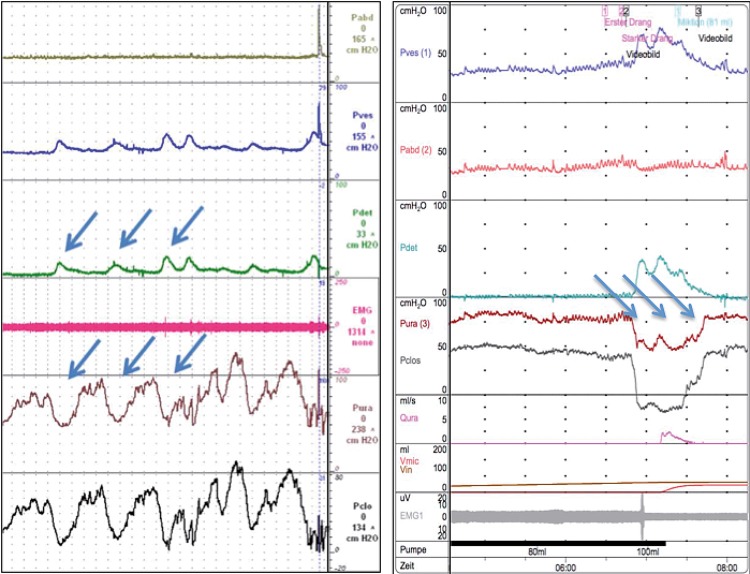
Urodynamic evaluation in a patient with DO (left) and SUI (right).

The prevalence of ‘urethral instability’ (pressure variance over 15cmH_2_O) in this cohort was 54.4% (43/79). For those with OAB syndrome, ‘urethral instability’ occurred in 23 of 29 (79.3%) patients, in 9 of 19 (47.4%) of those with stress incontinence and in 11 of 31 (36.6%) with mixed incontinence. Typical urodynamic traces of a patient with OAB and DO and a patient with SUI without DO are shown in Figure-1.

**Figure 2:**
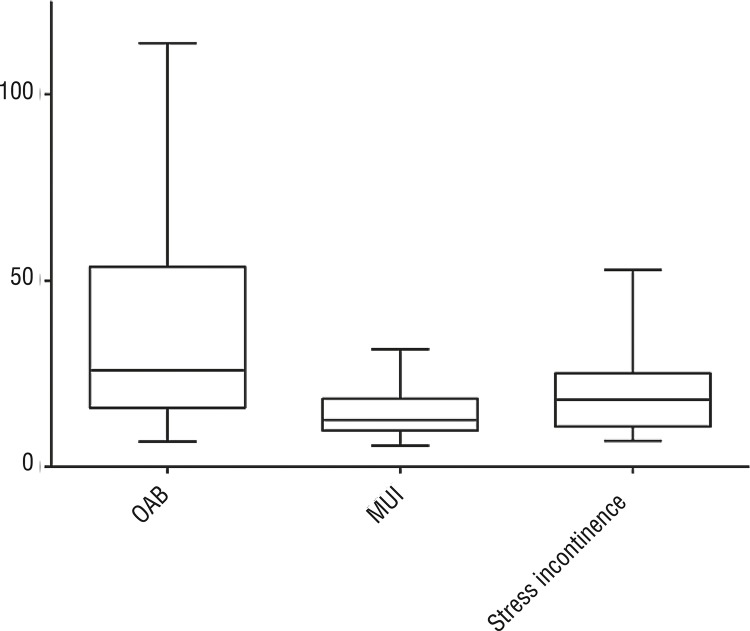
Diagram of variance of delta pressure between the groups.

**Figure 3:**
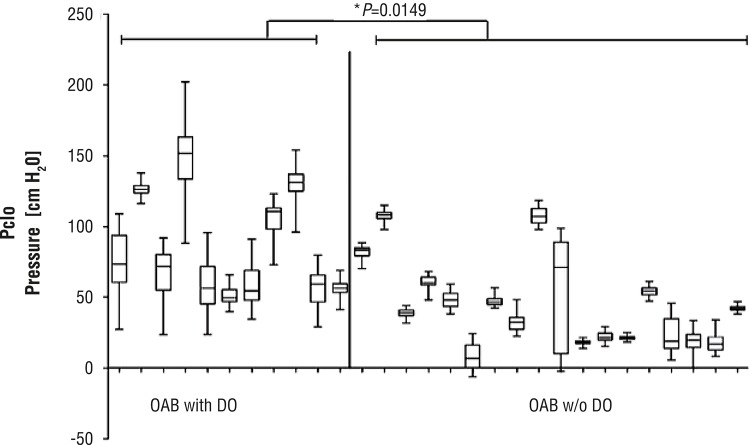
Urethral Pressure in OAB patients with (left) or without (right) DO, single asterisk displays significance difference between the two groups determined with two-tailed t-test.

The mean Δp (delta pressure) in patients with a sensory component related to OAB syndrome (36.5cmH_2_O) was significantly higher (p<0.05) than in groups with pure stress (14.9cmH_2_O) and mixed urinary incontinence (19.30cmH_2_O) (Figure-2). Patients of the OAB group were divided into sub-groups ‘with DO’ and ‘without DO’. Mean urethral pressure of OAB patients with DO (85.5±37.0cmH_2_O (mean±SD) was significantly higher than in OAB patients without DO (45.2±30.65cmH_2_O) (p<0.05) (Figure-3).

**Figure 4:**
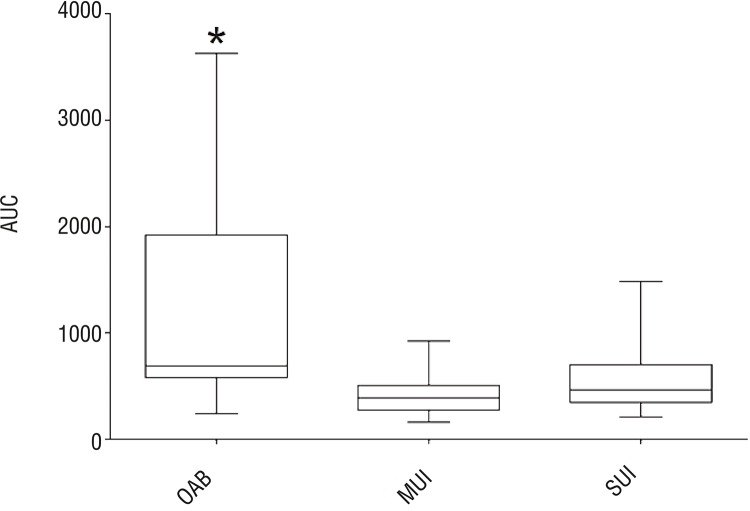
Area under the curve of cUPP in patients with OAB, MUI and SUI, applied trapezoidal rule calculated from min. pressure.

The analysis of the area under the curve showed that urethral pressure curves of OAB patients are in sum either longer or higher than those of mixed or stress urinary incontinence (1195.7±985.6 (OAB) vs. 429.3±214.5 (MUI) vs. SUI 549.4±273.0 (SUI) (Figure-4).

**Figure 5:**
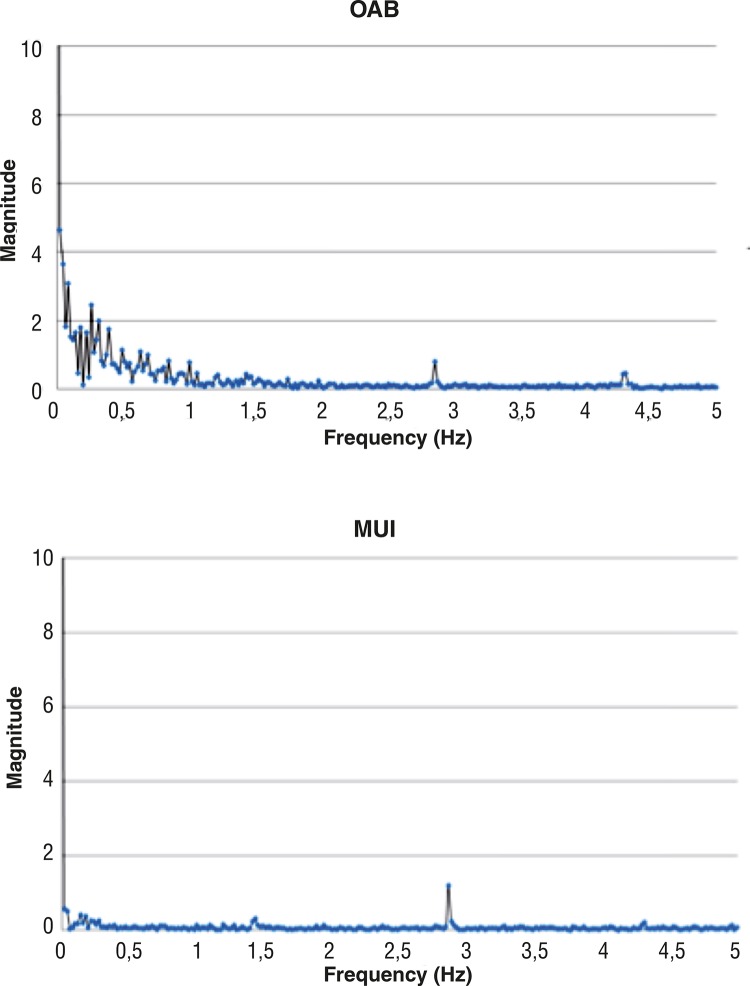
Representative FFT-Transformation of urethral pressure measures in a patient with OAB (above) and MUI (bottom).

Statistical analysis revealed significant differences with p<0.005 (OAB vs. MUI) and p<0.01 (OAB vs. SUI). Fast Fourier Transformation generated FFT-Plots for every patient and visual review revealed higher magnitudes and more peaks in the range of 0-0.5Hz within the group of patients with OAB (Figure-5).

## DISCUSSION

Our study revealed that patients with OAB have statistically significant higher range of urethral pressure variation than in SUI and MUI patient groups, moreover we found that in patients with OAB and concomitant DO urethral pressure variation range was even higher than without DO (p<0.05) (Figure-3).

During urodynamic investigations (UDS), the focus regularly lies on the bladder pressure changes neglecting the fact that in the process of urine storage, the external urethral sphincter may play an important role. In 1959, the “guarding reflex” by the external urethral sphincter was brought into discussion by Garry et al. and was attributed a main role in prohibiting stress incontinence (13). Referring to the “guarding reflex”, Park et al. later stipulated a theory that the external sphincter has a bladder-modulating role (4). McGuire and Sørensen in the 70s and 80s concluded that urethral pressure variations seem to play an important role in normal urethral physiology, possibly contributing to continence and prevention of urinary tract infection (14, 15). In contrast, Vereecken et al. failed to demonstrate a difference between patients with urge and stress incontinence with regard to ‘urethral instability’, except urethral pressure variations of more than 35cmH_2_O, which provoked urge (9). No difference in terms of prevalence or severity of urgency/frequency, nocturia or urge incontinence was reported between patients with or without ‘urethral instability’, and the symptom of stress incontinence was more common in women with ‘urethral instability’ (9, 16). Nevertheless, Matthiason et al. also came to the conclusion that women with stress, urge, and mixed urinary incontinence seem to have a primary neuromuscular disorder in the urethra. They described urethral pressure variation as an overactive opening mechanism with a fall in urethral pressure instead of a pressure increase on provocation during the filling phase of the bladder, and during bladder emptying a more efficient opening of the bladder outlet than in normal women. They suggested that one and the same pathophysiological mechanism participates in female stress, urge, and mixed incontinence (17). Moreover in 2009, Groenendijk et al. stated that it makes sense to measure and register detrusor and urethral function during filling and voiding (18). Despite the fact that most authors consider urethral pressure variations, exceeding an amplitude of 15cmH_2_O, as an abnormal finding (6-12) the significance of these fluctuations is still unclear, and the terms ‘urethral instability’ or ‘unstable urethra’ lack clarity and are not accurately defined by the ICS today.

In accordance with our own data, Sørensen et al. demonstrated that mean maximum urethral pressure and the mean maximum urethral closure pressure are significantly reduced in women with stress incontinence compared to women with detrusor overactivity (19).

Our study is not without limitations. First limitation is in the study design-small sample size, absence of assessors blinding, absence of a healthy patients group. Second is that the neurophysiologic explanation of urethral pressure variation is unclear. Urethral pressure variation may be caused by diminished sympathetic influence or by increased parasympathetic activity. It has been reported that neuronal nitric oxide (NO) synthase, known for its significant role in nociceptive pathways in the bladder has also been found in human female striated urethra sphincter (20). It may be that contraction of the detrusor is caused by a fall in urethral pressure (18). However, looking at the traces, it is very difficult to determine (Figure-1) which came first: detrusor overactivity, then urethral pressure drop or vice versa, or both appeared at the same time. Whatever concomitant finding of detrusor overactivity and urethral pressure variations may suggest a combined pathophysiology being a cofactor in some OAB patients. Considering that some DO might not have been detected, correlation should be even higher.

Further investigation of urethral pressure variation with high-speed urethral pressure urodynamics will let to sample at up to 1000Hz and not 10 to 50Hz used today in conventional equipment and possibly shed some more light on that issue.

## CONCLUSIONS

Given the growing relevance of OAB syndrome and widely expanding armamentarium of treatment modalities and drugs as well the outcomes are still quite disappointing (21) and it seems that we are missing something important in patients with OAB. Possibly we should stress our attention on both: detrusor overactivity and urethral instability.
